# Meteorin-like levels are associated with active brown adipose tissue in early infancy

**DOI:** 10.3389/fendo.2023.1136245

**Published:** 2023-03-02

**Authors:** Cristina Garcia-Beltran, Artur Navarro-Gascon, Abel López-Bermejo, Tania Quesada-López, Francis de Zegher, Lourdes Ibáñez, Francesc Villarroya

**Affiliations:** ^1^ Research Institute Sant Joan de Déu, University of Barcelona, Barcelona, Spain; ^2^ Centro de Investigación Biomédica en Red de Diabetes y Enfermedades Metabólicas Asociadas (CIBERDEM), Health Institute Carlos III, Madrid, Spain; ^3^ Biochemistry and Molecular Biomedicine Department, Biomedicine Institute, University of Barcelona, Barcelona, Spain; ^4^ Network Biomedical Research Center of Physiopathology of Obesity and Nutrition (CIBEROBN), Health Institute Carlos III, Madrid, Spain; ^5^ Department of Pediatrics, Dr. Josep Trueta Hospital, Girona, Spain; ^6^ Department of Medical Sciences, Faculty of Medicine, University of Girona, Girona, Spain; ^7^ Leuven Research and Development, University of Leuven, Leuven, Belgium

**Keywords:** meteorin-like (METRNL), brown adipose tissue (BAT), brown adipokine, infancy, thermogenesis

## Abstract

**Introduction:**

Meteorin-like (METRNL) is a hormonal factor released by several tissues, including thermogenically active brown and beige adipose tissues. It exerts multiple beneficial effects on metabolic and cardiovascular systems in experimental models. However, the potential role of METRNL as brown adipokine in humans has not been investigated previously, particularly in relation to the metabolic adaptations taking place in early life, when brown adipose tissue (BAT) is particularly abundant.

**Methods and materials:**

METRNL levels, as well as body composition (DXA) and circulating endocrine-metabolic variables, were assessed longitudinally in a cohort of infants at birth, and at ages 4 and 12 months. BAT activity was measured by infrared thermography at age 12 months. METRNL levels were also determined cross-sectionally in adults; *METRNL* gene expression (qRT-PCR) was assessed in BAT and liver samples from neonates, and in adipose tissue and liver samples form adults. Simpson-Golabi-Behmel Syndrome (SGBS) adipose cells were thermogenically activated using cAMP, and *METRNL* gene expression and METRNL protein released were analysed.

**Results:**

Serum METRNL levels were high at birth and declined across the first year of life albeit remaining higher than in adulthood. At age 4 and 12 months, METRNL levels correlated positively with circulating C-X-C motif chemokine ligand 14 (CXCL14), a chemokine released by thermogenically active BAT, but not with parameters of adiposity or metabolic status. METRNL levels also correlated positively with infrared thermography-estimated posterior-cervical BAT activity in girls aged 12 months. Gene expression analysis indicated high levels of *METRNL* mRNA in neonatal BAT. Thermogenic stimulus of brown/beige adipocytes led to a significant increase of *METRNL* gene expression and METRN protein release to the cell culture medium.

**Conclusion:**

Circulating METRNL levels are high in the first year of life and correlate with indices of BAT activity and with levels of an established brown adipokine such as CXCL14. These data, in addition with the high expression of *METRNL* in neonatal BAT and in thermogenically-stimulated brown/beige adipocytes, suggest that METRNL is actively secreted by BAT and may be a circulating biomarker of BAT activity in early life.

## 1 Introduction

Meteorin-like protein (METRNL), also known as Meteorin-β, interleukin-41 and subfatin, is a recently identified hormone involved in metabolic regulation and considered a candidate biomarker of metabolic syndrome (1). In rodent models, METRNL is highly expressed in brown adipose tissue (BAT) upon thermogenic activation and also in skeletal muscle after exercise (2). METRNL was found to promote energy expenditure and glucose tolerance through the induction of alternatively activated macrophages at adipose depots and by promoting the browning of adipose tissue. Further research showed that peroxisome proliferator–activated receptor-γ (PPARγ) enhances the capacity of METRNL to antagonize insulin resistance in adipose tissue (3). METRNL also attenuates inflammation and insulin resistance in skeletal muscle *via* AMP-activated protein kinase and PPARδ-dependent pathways (4, 5). The beneficial effects of METRNL have been associated with innate immunity (6, 7), and protection against cardiac dysfunction (8, 9). In adult humans, METRNL levels are low in patients with obesity and diabetes and correlate negatively with glucose levels and markers of insulin resistance (10–13).

Metabolic and nutritional alterations in the early postnatal life are not only relevant for health during infancy but may also contribute to the development of metabolic syndrome in later life. In recent years, the activity of thermogenic (brown/beige) adipose tissues in adult humans has gained attention as a protective factor against obesity, type 2 diabetes and cardiovascular disease (14). This is attributed to the capacity of BAT to both drain glucose and lipids for adaptive thermogenesis and to secrete adipokines with healthy effects on metabolism (15). However, despite the existing awareness that BAT size and activity are particularly relevant in infants (16), the pathophysiological consequences of distinct BAT activities early after birth have not been studied. The identification of BAT-derived adipokines in infants and their capacity to be used as biomarkers of metabolic health has also been scarcely undertaken, and only a few circulating molecules, such as bone morphogenetic protein-8B (BMP8B) and C-X-C motif chemokine ligand 14 (CXCL14), have respectively been associated with BAT activity in newborns and in one-year-old infants (17–19).

Here we determined for the first time the circulating levels of METRNL across the first year of life and disclosed a significant association between this variable and the extent of BAT activity.

## 2 Materials and methods

### 2.1 Study population and ethics

The primary study cohort consisted of 50 infants (27 girls and 23 boys) who were enrolled prenatally during the customary third trimester visit among Caucasian pregnant mothers consecutively seen in the outpatient clinics of Hospital Sant Joan de Déu and Hospital de Sant Boi – Parc Sanitari Sant Joan de Déu (Barcelona, Spain) (**Supplementary Figure 1**). These infants had previously participated in a longitudinal study assessing BAT activity and circulating levels of CXCL14 and BMP8B in the first year of life (18, 19).

Inclusion criteria were: maternally uncomplicated, singleton pregnancy with delivery at term (37-42 weeks), exclusive breastfeeding or formula-feeding in the first 4 months, postnatal follow-up completed (at 15 days, 4 and 12 months) and written informed consent. Exclusion criteria were maternal disease, alcohol or drug abuse, congenital malformations and complications at birth. Birth weight was not considered as inclusion or exclusion criterium; accordingly, the study population included infants with a wide range of birth weight Z-scores (between −2.9 and +1.0).

Circulating METRNL was exclusively measured in a subset of infants who had spare serum sample available at birth (20 girls and 18 boys), and at age 4 and 12 months (26 girls and 16 boys). Serum METRNL was also measured in 30 mothers of those infants (age, 33.6 ± 0.9 years) during the third trimester of pregnancy (**Supplementary Figure 1**). In addition, serum METRNL concentrations were analyzed cross-sectionally in healthy adult women (N= 10; age, 38.7 ± 1.9 years)

*METRNL* mRNA gene expression was assessed in dorso-interscapular BAT (N= 5) and liver (N= 6) post-mortem samples obtained on occasion of autopsies (2–3 h after the death) of Caucasian newborns with a gestational age of 28–36 weeks who survived, at most, 3 days post-partum, supplied by the Academy of Sciences of the Czech Republic as previously described (20) (**Supplementary Table 1**). For comparison, *METRNL* mRNA gene expression was also determined in adult liver samples (obtained from hepatic biopsies performed when a hepatic tumor was suspected, with a negative ultimate result), deltoid muscle samples (from adult individuals who underwent skeletal muscle biopsy because of muscle complaint in whom skeletal muscle histology was thereafter normal) and subcutaneous adipose tissue samples (obtained from volunteers), as described (8, 21).

The study was approved by the Institutional Review Board of the University of Barcelona, Sant Joan de Déu University Hospital; all participating mothers signed the informed consent at recruitment.

### 2.2 Clinical and endocrine-metabolic assessments

Maternal data were retrieved from hospital clinical records. Gestational age was calculated according to the last menses and validated by first-trimester ultrasound. Weight and length of the newborns were measured immediately after delivery, and again at age 4 months and 12 months.

Maternal venous samples were obtained during the third trimester of gestation, between week 28 and delivery. Neonatal blood samples were obtained at birth from the umbilical cord before placenta separation (22). At age 4 and 12 months, venous samples were obtained during the morning in the fasting state. Adult venous samples were also obtained after overnight fasting. The serum fraction of the samples was separated by centrifugation and stored at -80°C until analysis.

Serum glucose, insulin, insulin-like growth factor (IGF)-I, high-molecular-weight (HMW) adiponectin, CXCL14 and BMP8B were assessed as previously reported (18, 19). Serum METRNL levels were determined in serum and cell culture media with a specific human enzyme-linked immunosorbent assay kit [R&D Systems, Minneapolis, MN, USA; sensitivity: 0.64 ng/mL; intra-assay coefficient of variation (CV) <10%; inter-assay CV < 12%].

### 2.3 Body composition and BAT activity assessment

Body composition was assessed at age 15 days, 4 months and 12 months by dual-energy X-ray absorptiometry (DXA) with a Lunar Prodigy and Lunar software (version 3.4/3.5; Lunar Corp., Madison, WI, USA) adapted for infants (22).

As previously described (18), BAT activity at age 12 months was estimated through the infrared thermography-based measurement of the skin temperature overlying BAT depots. The parameters assessed included the maximal temperature at the posterior cervical (T_PCR_) and supraclavicular (T_SCR_) regions, and the extent of active BAT in these regions (Area_PCR_ and Area_SCR_).

### 2.4 Cell cultures of neonatal beige adipocytes

Pre-adipocyte cells obtained post-mortem from a 3-month-old infant with Simpson Golabi Behmel Syndrome (SGBS cells) (23), capable to differentiate into adipocytes bearing a beige phenotype (24, 25) were used. SGBS pre-adipocytes were maintained in Dulbecco’s modified Eagle’s (DMEM)/F12 medium, 10% fetal bovine serum (FBS). Beige adipogenic differentiation was initiated by incubating confluent cell cultures for 4 days in serum-free medium plus 20 nM insulin, 0.2 nM triiodothyronine, 100 nM cortisol, 25 nM dexamethasone, 500 µM 3-isobutyl-1-methyl-xanthine, and 2 µM rosiglitazone. Subsequently, cells were switched to DMEM/F12, 20 nM insulin, 0.2 nM triiodothyronine, and 100 nM cortisol and maintained for up to 10 days, when more than 90% cells have acquired differentiated adipocyte morphology. To induce thermogenic activation of adipocytes, differentiated cells were treated with 1mM dibutyril-cAMP for 24 hours. All cell culture reagents and drugs were from Sigma-Aldrich (St Louis, Missouri, USA). Cells were collected for RNA isolation and the cell culture medium, corresponding to 24 h before harvest, was also collected for measurement of METRNL levels.

### 2.5 RNA isolation and qRT-PCR analyses

RNA was extracted from tissues and cells using an affinity-based method (NucleoSpin, Macherey-Nagel, Germany). *METRNL, UCP1, PPARGC1A, DIO2 and BMP8B* transcript levels were determined by qRT-PCR using TaqMan technology (Thermo Fisher Scientific, Waltham, MA, USA). 0.5 μg RNA were retrotranscribed using random hexamer primers (Thermo Fisher Scientific, Waltham, MA, USA). For qRT-PCR, the *METRNL* (Hs00417150), *UCP1* (Hs00222453*), PPARGC1A* (Hs00173304), *DIO2* (Hs00255341), *BMP8B* (Hs01629120) TaqMan Gene Expression assay probes were used, with reaction mixtures containing 1 μL cDNA, 10 μL TaqMan Universal PCR Master Mix (Thermo Fisher Scientific, Waltham, MA, USA), 250 nM probes and 900 nM of primers from the Assays-on-Demand Gene Expression Assay Mix (Thermo Fisher Scientific, Waltham, MA, USA). The 18S rRNA transcript (Hs99999901) was measured as housekeeping reference gene. The mRNA level of *METRNL* and *UCP1*1: PGC1a, Dio2, Bmp8b in tissues and cells sample was normalized to that of the reference control using the comparative (2^–ΔCT^) method.

### 2.6 Statistics

Statistical analyses were implemented in SPSS version 27.0 (SPSS software, IBM, Armonk, NY, USA), GraphPad Prism 5 (GraphPad Software, CA, USA) and R Project version 4.2.2 (RStudio, MA, USA). Results are shown as mean ± standard error of the mean (SEM). Variables with normal distribution were compared with two-tailed Student’s t-test. Chi-square test was used to compare qualitative variables. Correlation and stepwise multi-regression analysis were used to study associations between circulating METRNL levels and the assessed variables; outliers were detected using a studentized residual outlier test and excluded from further analyses; this approach did not modify the statistical significance of any analysis. Covariance analysis was used to adjust for ponderal index and breastfeeding. A P-value < 0.05 was considered statistically significant.

## 3 Results

### 3.1 METRNL levels in the first year of life

**Supplementary Table 2** shows the longitudinal data from infants over the first year of life and from their mothers in late pregnancy in the cohort in which serum METRNL assessment was performed. As previously reported (18, 19), girls had less lean mass, higher levels of circulating CXCL14 and higher posterior BAT activity.

**Figure 1:**
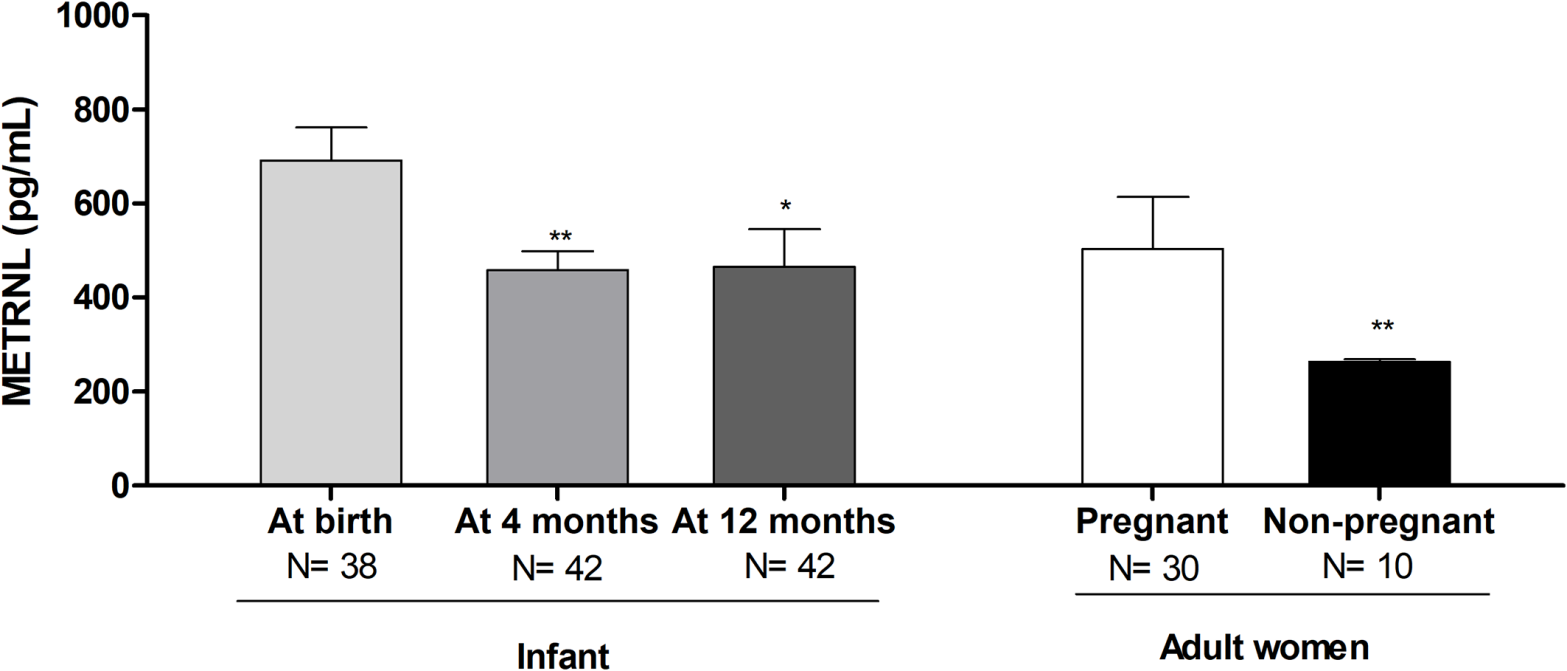
Serum Meteorin-like (METRNL) concentrations in human infants at birth and at age 4 and 12 months, in the mothers of those infants during the third trimester of pregnancy, and in healthy adult women. ^*^P<0.05, ^**^P<0.01 vs at birth. P values are adjusted for ponderal index and breastfeeding.

When splitting METRNL levels by sex, or by type of early feeding, no differences were found at any study time; accordingly, the results were pooled. Circulating METRNL concentrations in infants at birth were higher than at the postnatal ages of 4 and 12 months, and higher than in non-pregnant women (**Figure 1**).

### 3.2 Correlations between METRNL levels and clinical, endocrine-metabolic and body composition variables

**Figure 2:**
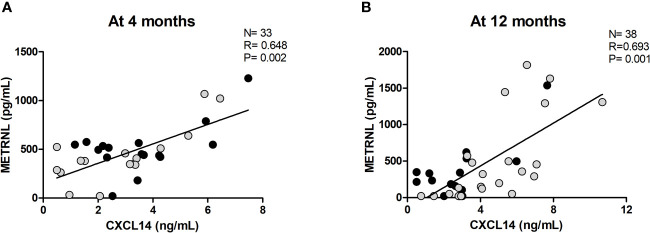
Correlation between circulating Meteorin-like (METRNL) and C-X-C motif chemokine ligand 14 (CXCL14) at age 4 months **(A)** and 12 months **(B)**. Grey dots correspond to girls and black dots represent boys. P values are adjusted for ponderal index and breastfeeding.

The associations between circulating levels of METRNL and anthropometric, adiposity-related, and endocrine-metabolic parameters, including some putative brown adipokines (CXCL14, BMP8B), throughout follow-up are shown in **Supplementary Table 3**. At birth, circulating METRNL levels were negatively related to abdominal fat only in girls (R= -0.678; P= 0.013, **Supplementary Table 3**). At age 4 months, circulating METRNL showed a strong positive correlation with circulating CXCL14 concentrations in the entire population (R= 0.648; P= 0.002) (**Figure 2A**). At age 12 months, METRNL concentrations were also positively correlated with CXCL14 levels (R= 0.693, P= 0.001) (**Figure 2B**). This correlation was maintained when girls were analyzed separately (R= 0.698; P= 0.012).

### 3.3 Correlations between METRNL concentrations and parameters of BAT activity

**Figure 3:**
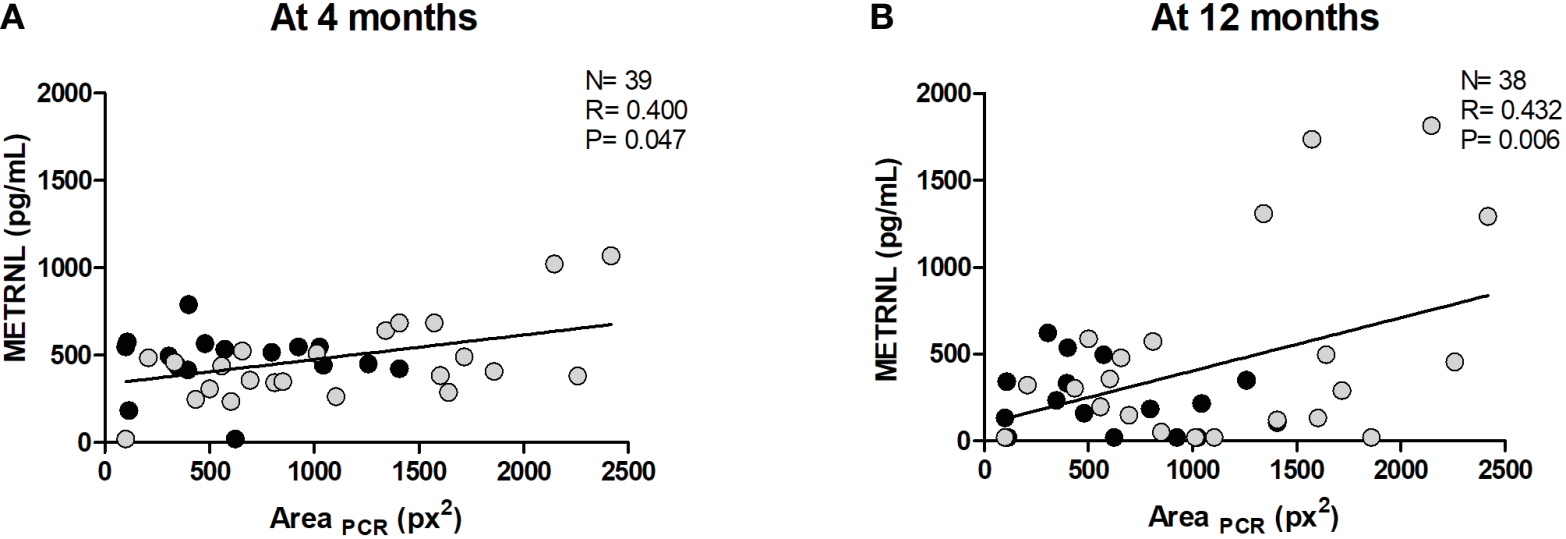
Correlation between the area of active brown adipose tissue at the posterior cervical region, as determined by infrared thermography (Area_PCR_), at age 12 months, and circulating Meteorin-like (METRNL) concentrations at age 4 months **(A)** and at age 12 months **(B)**. Grey dots correspond to girls and black dots represent boys. P values are adjusted for ponderal index and breastfeeding.

BAT activity at the posterior cervical and supraclavicular regions at age 12 months was analyzed in a subset of infants of the study cohort by using infrared thermography-based procedures, as previously described (18). Correlations between circulating METRNL levels at all time points and indicators of BAT activity at age 12 months are summarized in **Supplementary Table 4**. Significant positive correlations between posterior-cervical BAT activity and METRNL levels at 4 months (R= 0.400; P= 0.047; **Figure 3A**) and at 12 months (R= 0.432; P= 0.006; **Figure 3B**) were disclosed. Separate analyses by sex revealed a significant correlation between BAT activity and METRNL levels at 12 months only in girls (R= 0.426; P= 0.004).

### 3.4 *METRNL* expression in neonatal and adult tissues

**Figure 4:**
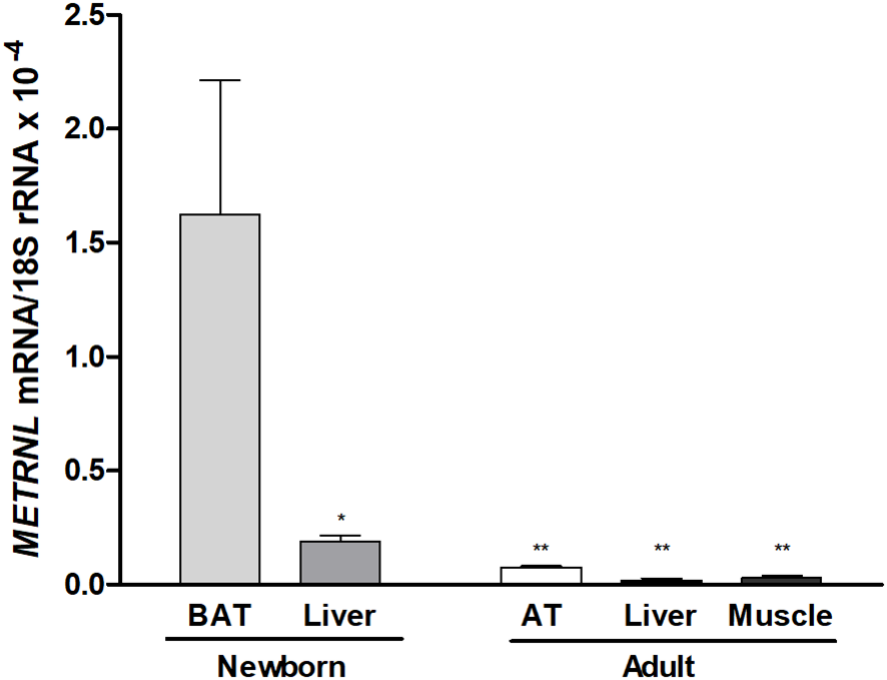
Meteorin-like (*METRNL*) gene expression levels in dorso-interscapular brown adipose tissue (BAT, N= 5) and liver (N= 6) obtained from post-mortem autopsies of newborns with a gestational age of 28–36 weeks who survived, at most, 3 days post-partum, and samples from subcutaneous adipose tissue (AT, N= 5), skeletal muscle (N = 5) and liver (N= 5) from healthy adults. Data are mean ± SEM of relative levels of *METRNL* mRNA (*METRNL* mRNA/18S rRNA). ^*^P<0.05; ^**^P<0.01 *vs* neonatal BAT.

*METRNL* expression levels in human neonatal post-mortem samples were significantly higher in dorso-interscapular BAT than in the liver. In addition, *METRNL* expression in neonatal BAT was much higher than in adult adipose tissue, skeletal muscle, and liver (**Figure 4**).

### 3.5 Activation of neonatal brown/beige adipocytes leads to increased *METRNL* expression and secretion of METRNL

**Figure 5:**
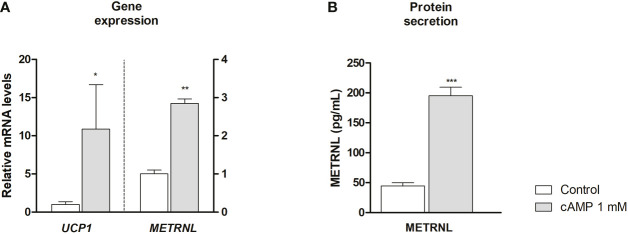
Meteorin-like (*METRNL*) gene expression **(A)** and METRNL protein secretion **(B)** by neonatal SGBS beige adipocytes. *METRNL* and uncoupling protein-1 (*UCP1*) transcript level **(A)** and METRNL protein levels **(B)** accumulating in cell culture medium after 24 h treatment of cell with 1 mM dibutyril-cAMP and untreated controls. Data mean ± SEM, N= 3 each condition. ^*^P<0.05; ^**^P<0.01; ^***^P<0.001 *vs* control.

Neonatal adipocytes differentiated into beige phenotype (SGBS cells) were thermogenically activated using cAMP (26). *METRNL* gene expression was dramatically induced, similarly to the thermogenic biomarker *UCP1* (**Figure 5A**) and other marker genes of brown/beige phenotype such as peroxisome proliferator-activated receptor-γ coactivator-1α (*PPARGC1A*), iodothyronine 5’-deiodinase (*DIO2*) and *BMP8B* (**Supplementary Figure 2**). Moreover, thermogenic activation of the cells also induced a significant increase in the release of METRNL protein to the culture medium (**Figure 5B**).

## 4 Discussion

The present study is, to our knowledge, the first to have assessed the circulating concentrations of METRNL in human infants, and to have related METRNL levels to BAT activity.

Our study demonstrates that circulating levels of METRNL – a novel adipokine that promotes browning of white adipocytes upon thermogenic stimulus (2) – is high at birth as compared to adult values and decreases over the first year of human life, although remaining higher than in adults. These findings are in line with those recently reported on circulating BMP8B – another brown adipokine involved in thermoregulation and metabolic homeostasis – that shows a similar decreasing trend from birth to age 12 months (19). Moreover, our data also disclosed a high expression of *METRNL* in neonatal human BAT and in thermogenic activated neonatal brown/beige adipocytes, as well as an increased secretion of METRNL in thermogenically activated cells, which confirms the capacity of human neonatal brown adipocytes to secrete this adipokine. Altogether, these data highlight the elevated activity of BAT after birth, when the demands for thermogenesis and risks for hypothermia can be especially high (16), and the concomitant production of BAT-secreted adipokines such as METRNL.

Circulating METRNL concentrations displayed a positive association with posterior-cervical BAT activity, as well as with circulating levels of CXCL14 – a chemokine secreted by active brown/beige adipose tissue (27). These data fit well with previous studies reporting a positive correlation between circulating CXCL14 levels and BAT activity in early life (18), and also between CXCL14 and METRNL expression in adult adipose tissue (28). Interestingly, CXCL14 and METRNL have emerged as circulating factors that modulate M2 macrophage activation playing a role in brown/beige thermogenic regulation (2, 27).

There is evidence of an interplay between BAT and skeletal muscle development in large mammalian species, which is characterized by a progressive decline in BAT after birth concomitant with skeletal muscle maturation, and this may affect BAT and muscle secretome (29). In rodents, skeletal muscle is a relevant site of *METRNL* gene expression (2) whereas in adult humans *METRNL* expression is low (8) but induced after exercise (2, 30). Lack of availability of muscle samples -or tissues other than BAT and liver- from neonates and young infants is a limitation of our study on *METRL* expression, and thus we cannot exclude a role of muscle or other tissues in influencing systemic METRNL levels in early development. Given the developmental overlap between BAT and muscle (29), it can’t be excluded that the correlation between METRNL levels and BAT activity in early infancy is indeed an indirect reflection of METRNL release by muscle. In any case, data retrieved from the transcriptomics database in muscle from infants in the first year of life and elderly adults does not indicate relevant differences in *METRNL* gene expression (31). Further studies would be required to establish the relative contribution of BAT and muscle to METRNL level changes in the first year of life.

Although METRNL levels were not different between girls and boys, the above-mentioned associations of METRNL levels in relation to BAT activity and CXCL14 levels at age 12 months were only maintained in girls. This finding may fit with the previously reported observation that BAT activity at that age is higher in girls than in boys (18) and with prior data reporting sex-based differences in the levels of other putative batokines such as CXCL14 and BMP8B (18, 19). There is extensive evidence of sex-based differences in BAT thermogenic activity due to direct and indirect hormonal mechanisms (32) and it is likely that sex-based differences occur also for the BAT secretome.

The distinct prevalence of “classic brown” versus “beige” adipocytes at specific anatomical BAT depots in humans (33, 34) may explain why circulating METRNL levels correlate with measures of posterior-cervical – but not supraclavicular – BAT activity. Differential secretory properties of brown-versus-beige cells have not yet been reported (even in experimental models) but a distinct capacity for METRNL secretion by different types of thermogenic adipocytes could account for the preferential association between METRNL levels and posterior-cervical BAT. On the other hand, high METRNL levels in early life, released by BAT and perhaps also by other tissues may promote a “browned” phenotype in white adipose depots in infants, given the known effects of METRNL in inducing the browning of adipose tissue (8), which would be especially adaptive to the thermally challenging conditions occurring in early infancy.

Circulating METRNL levels did not show significant correlations with systemic parameters of endocrine-metabolic status or adiposity in our cohort. This indicates that, although METRNL concentrations appear to be a potential indicator of the extent of BAT activity in one-year-old infants, they are poorly informative about their general endocrine-metabolic status. Possibly, the fact that our cohort involved apparently healthy children exhibiting no major differences in metabolic or adiposity parameters among individuals, precluded the identification of meaningful associations. Along these lines, the only significant correlation was the negative association between METRNL levels and abdominal fat in girls, present only at birth. Although this finding is reminiscent of the negative correlations between METRNL levels and visceral adiposity found in adults with obesity and/or type 2 diabetes (12, 13), the fact that it occurs only at birth indicates the need for future studies to explore a possible involvement of METRNL in the fat accretion occurring during late fetal development, something totally unknown to date.

Our study has several limitations, among them the relatively low number of serum samples available for METRNL assessment, the lack of tissue samples for *METRNL* mRNA gene expression from infants of the studied cohort due to obvious ethical reasons, and the lack of follow-up beyond age 1 year. Moreover, the lack of availability of neonatal samples from additional tissues (e.g. muscle) for gene expression analysis limited our capacity to infer whether, in addition to BAT, other tissue sources may be relevant contributors to systemic METRNL levels in infants, as in rodent models. Moreover, high levels of METRNL in blood from pregnant mothers, which may be caused by the high *METRNL* gene expression in placenta [data accessible at GEO profile database; GEO accession GDS3113, symbol 197624 (35)], may influence the high levels of METRNL in neonates at birth. On the other hand, further studies would be particularly interesting to assess in early infancy the potential relationship of METRNL levels with those of other secreted factors for which there is experimental evidence of involvement in BAT development, such as fibroblast growth factor (FGF)-9 or FGF21 (36). It should be also mentioned that our data on BAT activity were obtained by infrared methodology, which is minimally invasive but does not allow to assess the actual BAT mass which would require water-fat magnetic resonance imaging or quantification of the proton density fat fraction using magnetic resonance imaging (MRI) (37, 38). The strengths of our study include being the first assessment of METRNL levels in humans in early life and the co-availability of a large set of endocrine-metabolic, body composition and BAT activity data.

In summary, early life is associated with higher levels of circulating METRNL. The progressive reduction of METRNL concentrations in the first year of life -albeit maintained above those in adults- might reflect overall changes in BAT activity during early development. In addition, circulating METRNL concentrations associate with BAT activity and with CXCL14 levels, particularly in girls, supporting a role for METRNL as a brown adipokine and novel biomarker for BAT activity in early life.

## Data availability statement

The raw data supporting the conclusions of this article will be made available by the authors, without undue reservation.

## Ethics statement

The studies involving human participants were reviewed and approved by Institutional Review Board of the University of Barcelona, Sant Joan de Déu University Hospital, Spain. Written informed consent to participate in this study was provided by the participants’ legal guardian/next of kin.

## Author contributions

CG-B contributed to literature research, design of figures and tables, data collection, data analysis and interpretation. AN-G contributed to the analysis of circulating parameters and interpretation of data. TQ-L performed gene expression and cell culture-based studies. AL-B and FZ contributed to data interpretation, and reviewed/edited the manuscript. LI and FV contributed to study design, data interpretation, reviewed/edited the manuscript and wrote the manuscript. All authors contributed to the article and approved the submitted version.
