# Supplementary figures and images for: Meteorin-like levels are associated with active brown adipose tissue in early infancy
Source: Front Endocrinol (Lausanne). 2023 Mar 2;14:1136245. doi: 10.3389/fendo.2023.1136245 (PMC10018039; doi:10.3389/fendo.2023.1136245)

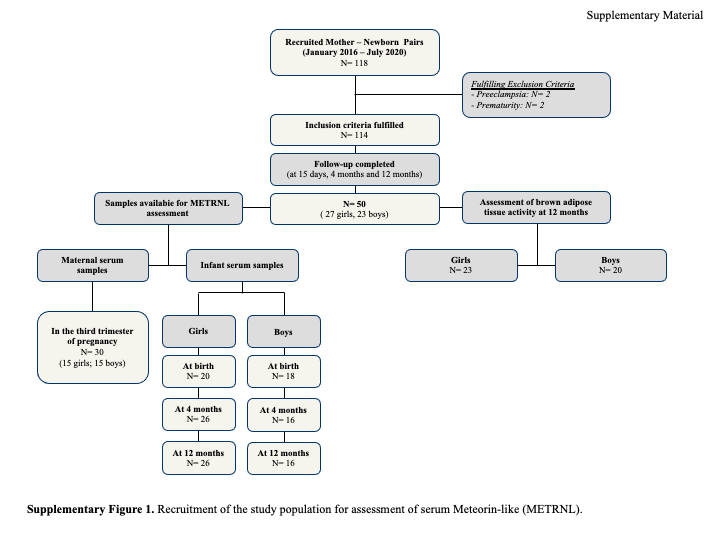

Supplement: Supplementary file 1 [file Image_1.tiff]

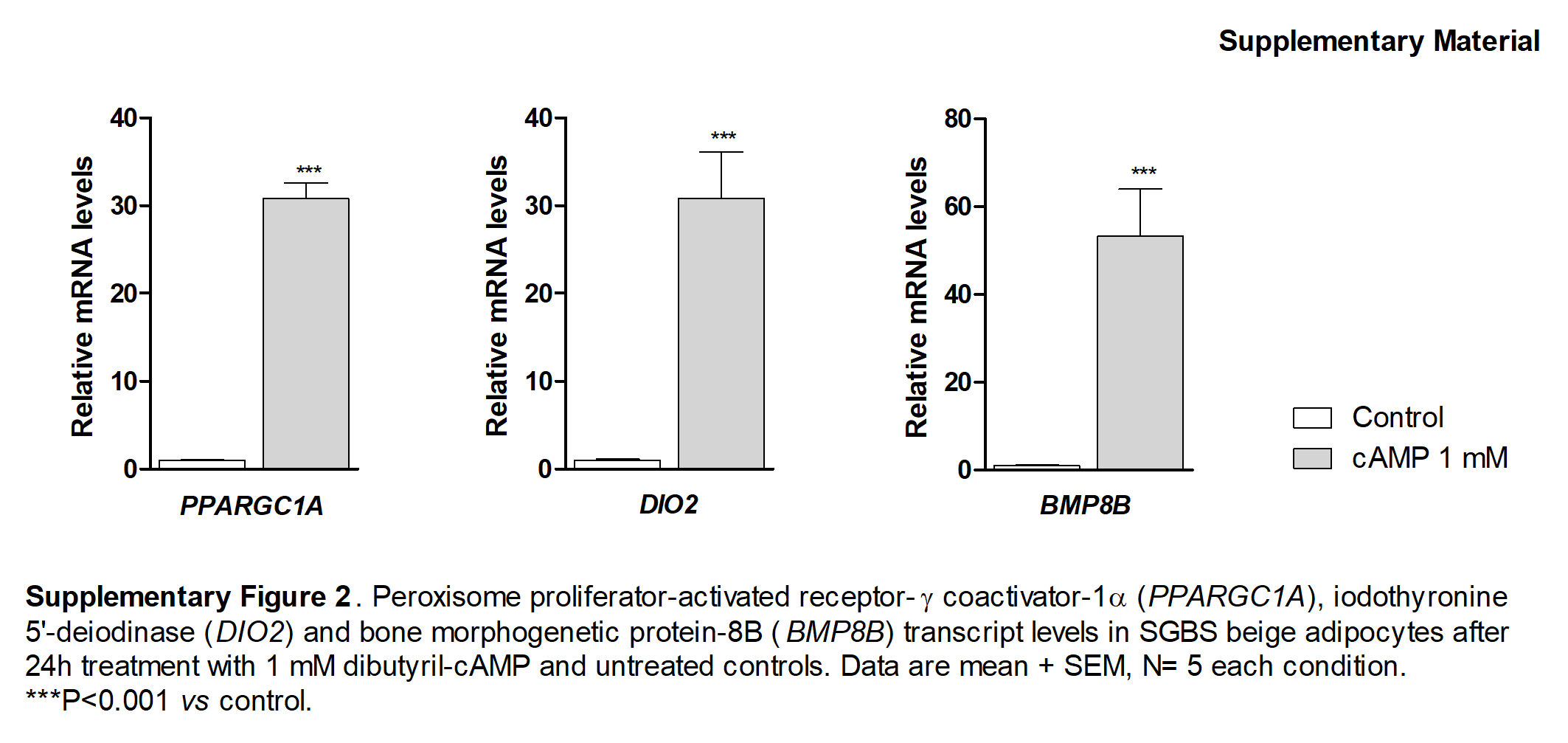

Supplement: Supplementary file 2 [file Image_2.tif]
