# Supplementary material for: Meteorin-like levels are associated with active brown adipose tissue in early infancy
Source: Front Endocrinol (Lausanne). 2023 Mar 2;14:1136245. doi: 10.3389/fendo.2023.1136245 (PMC10018039; doi:10.3389/fendo.2023.1136245)
Supplement: Supplementary file 3 [file Table_1.docx]

**Supplementary Table 1.**  Sex, gestational age, birth weight, survival time and clinical diagnosis of newborns whose brown adipose tissue and liver samples were included in the study of *Meteorin-like* (*METRNL*) gene expression.

| **Sex** | **Gestational age**  **(weeks)** | **Birth weight**  **(g)** | **Survival**  **(hours)** | **Clinical**  **diagnosis** |
| --- | --- | --- | --- | --- |
| F | 28 | 1050 | 0 | MM |
| M^*^ | 28 | 980 | 64 | PA, EOI, |
| M | 30 | 830 | 57 | ICH |
| M | 32 | 2060 | 41 | PH, RDS, ICH, IA, |
| M | 33 | 1840 | 10 | LH, RDS |
| M | 36 | 1860 | 3 | PNO, RA |

^*^only liver sample available. EOI, early onset infection; IA, intrauterine asphyxia; ICH, intracranial hemorrhage; LH, lung hypoplasia; MM, multiple malformations; PA, perinatal asphyxia; PH, pulmonary hemorrhage; PNO, pneumothorax; RA, renal agenesis; RDS, respiratory distress syndrome.
