# Supplementary material for: Meteorin-like levels are associated with active brown adipose tissue in early infancy
Source: Front Endocrinol (Lausanne). 2023 Mar 2;14:1136245. doi: 10.3389/fendo.2023.1136245 (PMC10018039; doi:10.3389/fendo.2023.1136245)
Supplement: Supplementary file 4 [file Table_2.docx]

**Supplementary Table 2.** Maternal and longitudinal infant data (from birth to age 12 months), from those infants from the primary cohort who had spare serum sample available for Meteorin-like (METRNL) assessment.

|  | **At birth** | | **At 4 months** | | **At 12 months** | |
| --- | --- | --- | --- | --- | --- | --- |
|  | **Girls**  **(N= 20)** | **Boys**  **(N= 18)** | **Girls**  **(N= 26)** | **Boys**  **(N= 16)** | **Girls**  **(N= 26)** | **Boys**  **(N= 16)** |
| **MOTHERS ^a^** | | | | | | |
| Age (years) | 32.5 ± 1.1 | 34.6 ± 1.2 | - | - | - | - |
| Pre-gestational weight (kg) | 61.1 ± 2.9 | 60.6 ± 3.7 | - | - | - | - |
| Pre-gestational BMI (kg/m^2^) | 24.2 ± 1.3 | 23.4 ± 1.3 | - | - | - | - |
| Primiparous (N) | 8 | 9 | - | - | - | - |
| Cesarean section (N) | 4 | 5 | - | - | - | - |
| Smoking (N) | 1 | 2 | - | - | - | - |
| **INFANTS** | | | | | | |
| **Anthropometry** | | | | | | |
| Gestational age (weeks) | 38.9 ± 0.3 | 38.4 ± 0.4 | - | - | - | - |
| Breastfeeding (N, %) | 14, 70% | 12, 66.7% | - | - | - | - |
| Weight (kg) | 2.8 ± 0.1 | 2.8 ± 0.2 | **5.8 ± 0.2** | **6.5 ± 0.2^*^** | 8.7 ± 0.2 | 8.9 ± 0.5 |
| Length (cm) | 48.1 ± 0.5 | 47.8 ± 0.9 | **59.4 ± 0.8** | **61.2 ± 1.0^*^** | 71.7 ± 0.9 | 72.1 ± 1.4 |
| BMI (Kg/m^2^) | 11.9 ± 0.2 | 11.8 ± 0.4 | **16.3 ± 0.4** | **17.2 ± 0.3^*^** | 16.8 ± 0.4 | 17.2 ± 1.1 |
| **Body composition (DXA) ^b^** | | | | | | |
| BMD (g/cm^2^) | 0.25 ± 0.01 | 0.23 ± 0.02 | 0.27 ± 0.01 | 0.27 ± 0.01 | 0.34 ± 0.01 | 0.34 ± 0.01 |
| Fat mass (kg) | 0.60 ± 0.04 | 0.64 ± 0.09 | 2.40 ± 0.11 | 2.29 ± 0.12 | 3.30 ± 0.14 | 3.68 ± 0.38 |
| Abdominal fat (kg) | 0.02 ± 0.00 | 0.03 ± 0.01 | 0.14 ± 0.01 | 0.14 ± 0.01 | 0.18 ± 0.02 | 0.17 ± 0.02 |
| Lean mass (kg) | 2.53 ± 0.11 | 2.69 ± 0.17 | **4.14 ± 0.10** | **4.58 ± 0.14^*^** | **6.15 ± 0.14** | **6.90 ± 0.18^***^** |
| **Endocrine-metabolic variables** | | | | | | |
| Glucose (mmol/L) | - | - | 4.9 ± 0.1 | 4.8 ± 0.1 | 4.5 ± 0.1 | 4.6 ± 0.1 |
| Insulin (pmol/L) | - | - | 37.3 ± 6.6 | 33.6 ± 8.5 | 57.8 ± 21.7 | 21.0 ± 6.5 |
| IGF-I (µg/L) | - | - | 34.9 ± 6.6 | 37.2 ± 4.2 | 59.3 ± 4.5 | 50.4 ± 6.1 |
| HMW-adip (mg/L)^c^ | - | - | 28.5 ± 2.5 | 29.6 ± 2.0 | 13.0 ± 0.7 | 14.8 ± 2.2 |
| CXCL14 (ng/mL)^c^ | - | - | 3.5 ± 1.1 | 3.5 ± 0.4 | **4.9 ± 0.5** | **2.9 ± 0.5^*^** |
| BMP8B (pg/mL) | - | - | 1011 ± 90 | 804 ± 122 | 509 ± 50 | 539 ± 84 |
| **Assessment of BAT activity ^d^** | | | | | | |
| T_PCR_ (^o^C) | - | - | - | - | 35.6 ± 0.1 | 35.7 ± 0.1 |
| T_PCR_ - T_SK_ (^o^C) | - | - | - | - | **1.3 ± 0.1** | **0.9 ± 0.1^*^** |
| Area_PCR_ (px^2^) | - | - | - | - | **1080.1 ± 143.1** | **653.5 ± 104.4^*^** |
| T_SCR_ (^o^C) | - | - | - | - | 35.6 ± 0.1 | 35.8 ± 0.1 |
| T_SCR_ - T_SK_ (^o^C) | - | - | - | - | 1.2 ± 0.1 | 1.2 ± 0.1 |
| Area_SCR_ (px^2^) | - | - | - | - | 571.9 ± 87.5 | 613.0 ± 124.8 |

BMI, body mass index; DXA, dual-energy X-ray absorptiometry; BMD, bone mineral density; IGF-I, insulin-like growth factor-I; HMW-adip, high-molecular-weight adiponectin; CXCL14, C-X-C motif chemokine ligand 14; BMP8B, bone morphogenetic protein 8-B; BAT, brown adipose tissue; PCR, posterior cervical region; SCR, supraclavicular region.

^a^ N= 30 women in the third trimester of pregnancy delivering girls (N= 15) and boys (N= 15); ^b^ at age 15 days instead of at birth; ^c^ at age 4 months, CXCL14 and HMW-adip assessments were performed in 17 out of 26 girls and at age 12 months, were performed in 22 out of 26 girls; ^d^ BAT activity was assessed in 23 out of 26 girls.

Statistically significant values are in bold. Values are mean ± SEM

^*^P<0.05, ^**^P<0.01 and ^***^P<0.001 vs girls at birth, at age 4 and 12 months. P values are adjusted for ponderal index and breastfeeding.
