# Supplementary material for: Meteorin-like levels are associated with active brown adipose tissue in early infancy
Source: Front Endocrinol (Lausanne). 2023 Mar 2;14:1136245. doi: 10.3389/fendo.2023.1136245 (PMC10018039; doi:10.3389/fendo.2023.1136245)
Supplement: Supplementary file 5 [file Table_3.docx]

**Supplementary Table 3.** Association between circulating Meteorin-like (METRNL) levels and clinical, endocrine-metabolic and body composition variables.

|  | **METRNL (pg/mL) at birth** | | | | | |
| --- | --- | --- | --- | --- | --- | --- |
|  | **All (N= 38)^*^** | | **Girls (N= 20)** | | **Boys (N= 18)^*^** | |
| **At birth** | **R** | **P** | **R** | **P** | **R** | **P** |
| Weight (kg) | -0.265 | 0.118 | -0.574 | 0.083 | -0.272 | 0.289 |
| Length (cm) | -0.179 | 0.578 | -0.7311 | 0.099 | 0.533 | 0.447 |
| BMI (kg/cm^2^) | -0.207 | 0.519 | -0.777 | 0.069 | 0.410 | 0.590 |
| BMD (g/cm^2^) ^a^ | 0.237 | 0.458 | -0.059 | 0.912 | 0.819 | 0.181 |
| Fat mass (kg) ^a^ | -0.069 | 0.831 | -0.737 | 0.095 | 0.772 | 0.228 |
| Abdominal fat (kg) ^a^ | -0.048 | 0.881 | **-0.675** | **0.013** | 0.612 | 0.388 |
| Lean mass (kg) ^a^ | -0.201 | 0.531 | -0.785 | 0.065 | 0.294 | 0.706 |
|  |  |  |  |  |  |  |
|  | **METRNL (pg/mL) at 4 months** | | | | | |
|  | **All (N= 42)** | | **Girls (N= 26)** | | **Boys (N= 16)** | |
| **At 4 months** | **R** | **P** | **R** | **P** | **R** | **P** |
| Weight (kg) | 0.139 | 0.558 | -0.574 | 0.097 | -0.206 | 0.543 |
| Length (cm) | 0.131 | 0.583 | -0.651 | 0.113 | -0.232 | 0.493 |
| BMI (kg/cm^2^) | 0.149 | 0.531 | -0.678 | 0.094 | -0.188 | 0.581 |
| BMD (g/cm^2^) | 0.343 | 0.140 | 0.118 | 0.800 | 0.388 | 0.238 |
| Fat mass (kg) | -0.145 | 0.541 | -0.728 | 0.063 | -0.234 | 0.489 |
| Abdominal fat (kg) | -0.098 | 0.680 | -0.385 | 0.394 | -0.230 | 0.496 |
| Lean mass (kg) | 0.248 | 0.292 | -0.868 | 0.071 | -0.009 | 0.979 |
| Glucose (mmol/L) | 0.043 | 0.856 | -0.417 | 0.352 | 0.154 | 0.654 |
| Insulin (pmol/L) | -0.242 | 0.304 | -0.030 | 0.949 | -0.451 | 0.158 |
| IGF-I (µg/L) | -0.166 | 0.483 | 0.466 | 0.291 | -0.259 | 0.442 |
| HMW-adip (mg/L) ^b^ | 0.149 | 0.530 | 0.706 | 0.077 | 0.142 | 0.677 |
| CXCL14 (ng/mL) ^b^ | **0.648** | **0.002** | 0.569 | 0.183 | 0.542 | 0.085 |
| BMP8B (pg/mL) | -0.225 | 0.339 | 0.680 | 0.093 | -0.202 | 0.551 |
|  |  |  |  |  |  |  |
|  | **METRNL (pg/mL) at 12 months** | | | | | |
|  | **All (N= 42)^#^** | | **Girls (N= 26)** | | **Boys (N= 16)^#^** | |
| **At 12 months** | **R** | **P** | **R** | **P** | **R** | **P** |
| Weight (kg) | 0.010 | 0.961 | 0.268 | 0.400 | -0.031 | 0.928 |
| Length (cm) | -0.110 | 0.484 | 0.305 | 0.336 | -0.024 | 0.925 |
| BMI (kg/cm^2^) | 0.120 | 0.447 | 0.266 | 0.404 | -0.132 | 0.612 |
| BMD (g/cm^2^) | -0.196 | 0.347 | -0.331 | 0.294 | -0.004 | 0.991 |
| Fat mass (kg) | -0.102 | 0.518 | -0.215 | 0.503 | -0.156 | 0.533 |
| Abdominal fat (kg) | -0.120 | 0.569 | -0.227 | 0.478 | -0.305 | 0.363 |
| Lean mass (kg) | -0.009 | 0.967 | 0.524 | 0.080 | 0.305 | 0.362 |
| Glucose (mmol/L) | 0.035 | 0.867 | -0.100 | 0.758 | 0.418 | 0.201 |
| Insulin (pmol/L) | -0.204 | 0.909 | -0.292 | 0.358 | 0.093 | 0.785 |
| IGF-I (µg/L) | 0.201 | 0.334 | 0.403 | 0.194 | -0.132 | 0.699 |
| HMW-adip (mg/L) ^c^ | -0.152 | 0.468 | -0.256 | 0.423 | -0.237 | 0.484 |
| CXCL14 (ng/mL) ^c^ | **0.693** | **0.001** | **0.698** | **0.012** | 0.176 | 0.605 |
| BMP8B (pg/mL) | 0.093 | 0.658 | 0.428 | 0.165 | -0.295 | 0.379s |

BMI, body mass index; BMD, bone mineral density; IGF-I, insulin-like growth factor-I; HMW-adip, high-molecular-weight adiponectin; CXCL14, C-X-C motif chemokine ligand 14; BMP8B, bone morphogenetic protein 8-B.

^a^At age 15 days instead of at birth. ^b^At age 4 months, CXCL14 and HMW-adip assessment was performed in 17 out of 26 girls. ^c^At age 12 months, CXCL14 and HMW-adip assessment was performed in 22 out of 26 girls.

^*^R and P values for associations between circulating METRNL and birth weight, and those between circulating METRNL and length, BMI and fat mass are shown after exclusion of a single outlier.

Results are shown as R coefficients and P values, adjusted for ponderal index and breastfeeding in multiple regression analysis. Statistically significant values are in bold.
