# Supplementary material for: Meteorin-like levels are associated with active brown adipose tissue in early infancy
Source: Front Endocrinol (Lausanne). 2023 Mar 2;14:1136245. doi: 10.3389/fendo.2023.1136245 (PMC10018039; doi:10.3389/fendo.2023.1136245)
Supplement: Supplementary file 6 [file Table_4.docx]

**Supplementary Table 4.** Association between circulating Meteorin-like (METRNL) levels and indicators of brown adipose tissue activity.

|  | **METRNL (pg/mL) at birth** | | | | | |
| --- | --- | --- | --- | --- | --- | --- |
|  | **All (N= 38)** | | **Girls (N= 20)** | | **Boys (N= 18)** | |
| **At 12 months** | **R** | **P** | **R** | **P** | **R** | **P** |
| T_PCR_ (^o^C) | -0.265 | 0.117 | -0.373 | 0.466 | -0.678 | 0.322 |
| T_PCR_ - T_SK_ (^o^C) | -0.256 | 0.423 | -0.418 | 0.409 | 0.144 | 0.886 |
| Area _PCR_ (px^2^) | -0.667 | 0.079 | -0.709 | 0.115 | -0.881 | 0.119 |
| T_SCR_ (^o^C) | -0.227 | 0.477 | -0.091 | 0.864 | -0.861 | 0.139 |
| T_SCR_ - T_SK_ (^o^C) | -0.259 | 0.431 | 0.460 | 0.358 | -0.827 | 0.173 |
| Area _SCR_ (px^2^) | -0.596 | 0.051 | -0.577 | 0.231 | -0.843 | 0.157 |
|  | | | | | | |
|  | **METRNL (pg/mL) at 4 months** | | | | | |
|  | **All (N= 39)** | | **Girls (N= 23)** | | **Boys (N= 16)** | |
| **At 12 months** | **R** | **P** | **R** | **P** | **R** | **P** |
| T_PCR_ (^o^C) | 0.000 | 0.998 | 0.221 | 0.489 | -0.499 | 0.118 |
| T_PCR_ - T_SK_ (^o^C) | 0.231 | 0.266 | 0.337 | 0.285 | 0.133 | 0.696 |
| Area _PCR_ (px^2^) | **0.400** | **0.047** | 0.555 | 0.056 | 0.347 | 0.297 |
| T_SCR_ (^o^C) | -0.177 | 0.399 | -0.071 | 0.826 | -0.359 | 0.278 |
| T_SCR_ - T_SK_ (^o^C) | -0.072 | 0.732 | 0.130 | 0.688 | -0.283 | 0.398 |
| Area _SCR_ (px^2^) | 0.065 | 0.756 | 0.425 | 0.168 | 0.062 | 0.855 |
|  | | | | | | |
|  | **METRNL (pg/mL) at 12 months** | | | | | |
|  | **All (N= 39)^*^** | | **Girls (N= 23)^*^** | | **Boys (N= 16)** | |
| **At 12 months** | **R** | **P** | **R** | **P** | **R** | **P** |
| T_PCR_ (^o^C) | -0.160 | 0.414 | 0.301 | 0.315 | -0.314 | 0.099 |
| T_PCR_ - T_SK_ (^o^C) | 0.030 | 0.882 | 0.054 | 0.860 | -0.008 | 0.981 |
| Area _PCR_ (px^2^) | **0.432** | **0.006** | **0.426** | **0.004** | 0.321 | 0.309 |
| T_SCR_ (^o^C) | -0.275 | 0.165 | 0.008 | 0.979 | -0.392 | 0.093 |
| T_SCR_ - T_SK_ (^o^C) | -0.101 | 0.615 | 0.012 | 0.969 | -0.199 | 0.536 |
| Area _SCR_ (px^2^) | 0.185 | 0.355 | 0.283 | 0.348 | 0.226 | 0.480 |

PCR, posterior cervical region; SCR, supraclavicular region.

^*^ R and P values for correlation between circulating METRNL and the Area _PCR_ are shown after exclusion of a single outlier value.

Results are shown as R coefficients and P values, adjusted for ponderal index and breastfeeding in multiple regression analysis. Statistically significant values are in bold.
